# Human immunodeficiency virus and hepatitis B genotype G/A2 recombinant co-infection: a case study

**DOI:** 10.1186/s40064-016-3169-2

**Published:** 2016-09-07

**Authors:** Eisuke Adachi, Masaya Sugiyama, Sayaka Shimizu, Kako Kodama, Tadashi Kikuchi, Michiko Koga, Masashi Mizokami, Tomohiko Koibuchi

**Affiliations:** 1 Department of Infectious Diseases and Applied Immunology, IMSUT Hospital of The Institute of Medical Science, The University of Tokyo, 4-6-1 Shirokanedai, Minato-ku, Tokyo, 108-8639 Japan; 2 Genome Medical Science Project, The Research Center for Hepatitis and Immunology, National Center for Global Health and Medicine, 1-7-1 Kohnodai, Ichikawa-shi, Chiba 272-8516 Japan; 3 Department of Hepatology Center, Kitasato University Kitasato Institute Hospital, 5-9-1 Shirokane, Minato-ku, Tokyo, 108-8642 Japan; 4 Department of Rheumatology and Infectious Diseases, Kitasato University School of Medicine, 1-15-1 Kitasato, Minami-ku, Sagamihara-shi, Kanagawa 252-0375 Japan

**Keywords:** Hepatitis B virus, Human immunodeficiency virus, Recombinants with HBV genotype G, Antiretroviral therapy

## Abstract

**Background:**

Hepatitis B virus (HBV) genotypes have distinct geographical distributions and are associated with different clinical courses. HBV genotype G (HBV/G) is extremely rare among Human immunodeficiency virus (HIV) infected populations in Japan. Genetic analysis and clinical course of recombinant forms with HBV/G infection are seldom reported in the literature.

**Case presentation:**

A 36-year old homosexual man with HIV infection was referred to a general hospital for assessment of chronic HBV infection. We cloned full-length HBV isolates and determined the complete genome sequences of 2 obtained clones, although mixture of multiple variant with different length is detected by HBV-DNA genotyping. The Bootscaning analysis using a full-length HBV genome revealed the clones represented as the HBV/A2 and the HBV/G/A2 recombinant strain. The HBV-DNA decreased from >9.1 to 2.5 log copies/mL after 24 months of antiretroviral therapy.

**Conclusions:**

This patient was co-infected with HBV/A2 and HBV/G/A2 recombinant strain. This recombinant strain was not identical to HBV/G/A2 strains previously reported from Japan. Recombination with other genotypes could alter the clinical manifestations of chronic hepatitis B in people living with HIV.

## Background

Hepatitis B virus (HBV) infection is a major public health issues among human immunodeficiency virus (HIV) -infected people. HIV-HBV co-infection can accelerate the progression of chronic hepatitis resulting in fibrosis and hepatocelullar carcinoma (Thio et al. 2002). HIV-HBV co-infection rate is high due to the similar transmission route and approximately 6.3 % of the HIV-infected patients in Japan are coinfected with HBV (Koike et al. 2008).

HBV is classified into ten genotypes on the basis of 8 % divergence in the entire genomic sequence. HBV genotypes have distinct geographical distributions and are associated with different clinical courses. In HIV-HBV coinfected patients in Japan, HBV genotype A2 (HBV/A2) is the most frequently detected, followed far behind by genotype C and genotype B which are common in the entire chronic hepatitis B population (Yanagimoto et al. 2012). HBV genotype G (HBV/G) was first reported in 2000 from France and characterized by a unique 36 nt insertion in the core region and the possession of two stop codons in the precore region that prevents the expression of hepatitis B envelope antigen (HBeAg) (Stuyver et al. 2000). HBV/G infections were found to occur predominantly in males (92 %) and were primarily associated with male homosexual sex (67 %) in Canada (Osiowy et al. 2008). French studies found a high prevalence of patients infected with HBV/G among HIV-HBV co-infected patients (12 and 25 %) (Lacombe et al. 2006; Desire et al. 2010). In contrast, HBV/G is extremely rare in Japan even in HIV-infected patients (Yanagimoto et al. 2012; Kato et al. 2004). One of its unique characteristics is frequent co-infection with the other genotypes. Recombinant forms between HBV/G and the other genotypes have been observed. However, these recombinant forms have been detected normally as a minority species within the HBV quasispecies population and specific prevalent strains have not been identified (Kato et al. 2002a, b). Some epidemiological data exhibited patients infected with HIV-HBV/G were at increased risk of liver fibrosis compared to those infected with other HBV genotypes (Lacombe et al. 2006; Dao et al. 2011) but clinical data on HBV/G is limited due to the low prevalence throughout the world. We report here a case of chronic hepatitis caused by HIV-HBV/G/A2 recombinant strain co-infection. These epidemiological and clinical findings have warranted the need for HBV genotyping in the management of HIV-HBV co-infected patients.

## Case presentation

**Fig. 1:**
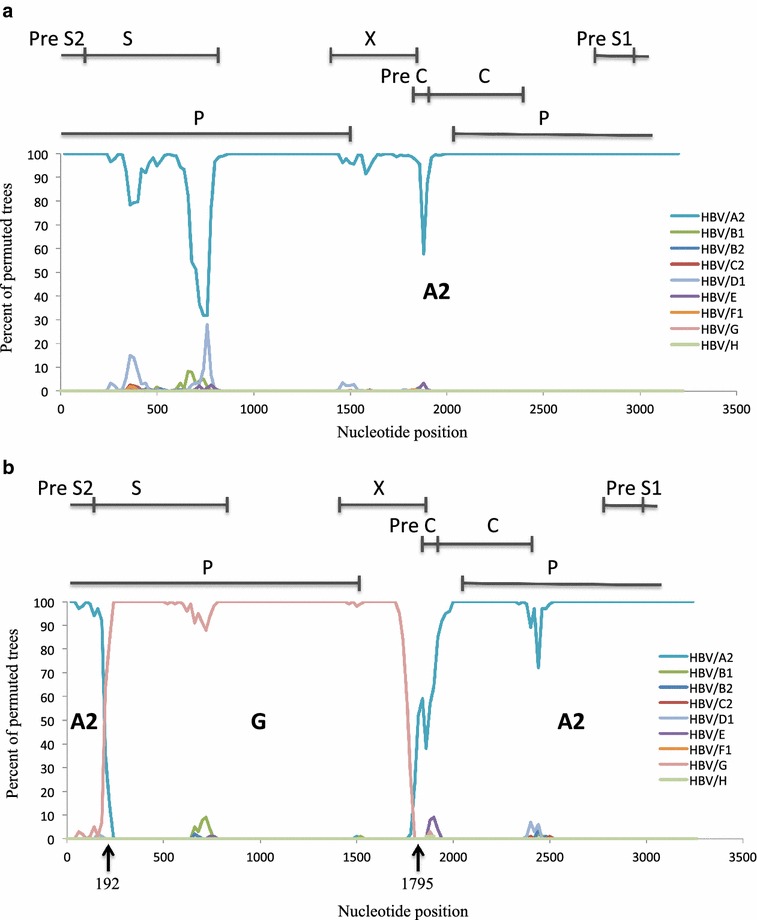
Bootscaning analysis with the following parameters: a window size of 200 bp, a step size of 20 bp, 1000 bootstrap replicates, gapstrip on and neighbor-joining analysis by RDP4. **a** HBV genotype A2 **b** HBV genotype G and A2 recombinant. The *black arrows* indicate the break points of genomic recombination

**Fig. 2:**
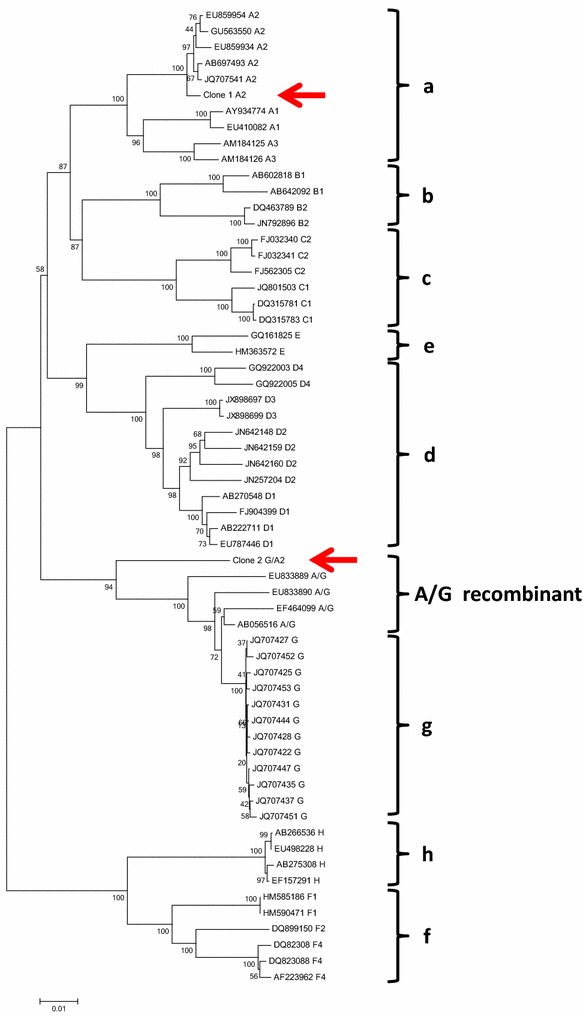
Phylogenetic analysis. The *red arrows* indicate the sequences in this case: clone 1 A2, HBV/A2 (Fig. 1a). clone 2 G/A2, HBV/G/A2 recombinant (Fig. 1b)

In 2013, a 36 year-old Japanese man was diagnosed with chronic hepatitis B based on the elevated levels of liver enzyme and the positive hepatitis B surface antigen (HBsAg) at a local clinic during follow-up for flu-like symptoms. He was referred to the hepatology division, in a general hospital for further assessment of chronic hepatitis B. The liver biopsy showed septal fibrosis and infiltrating lymphocyte in his liver tissue associated with degeneration of limiting plate, and histologic activity index—METAVIR score (Thio et al. 2002) was A2-F3. He had been living in Japan all his life and had no family histories of viral hepatitis. He denied intravenous drug use and dissolute sexual behavior in Japan, but admitted occasional sexual intercourse with men in Thailand since he was 20 years old. The screening for HIV antigen/antibody test was positive and HIV infection was confirmed by the positive Western-blot HIV antibody test. The Serological HBV genotyping test by enzyme immunoassay (EIA) in the clinical laboratory testing industry (SRL Inc. Japan) showed genotype D. However, his social history was not compatible with the risk of chronically HBV gonotype D (HBV/D) infection because HBV/D is rare throughout Japan except for some areas and not prevalent among men who have sex with men (MSM). Then, HBV-DNA genotyping was performed using obtained isolates from this patient. We cloned full-length HBV isolates and determined the complete genome sequences of 2 obtained clones as previously reported (Sugauchi et al. 2001). Briefly, the full length of HBV genome was amplified as two overlapping fragments by using the primer set to yield a 3200 bp amplicon for fragment A and another primer set to yield a 462 bp amplicon for fragment B. The fragment A or B was transferred to pGEM-T easy vector by TA cloning. The cloned plasmids of fragment A or B were sequenced and analyzed by phylogenetic analysis using MEGA6 (Tamura et al. 2013). The Bootscaning analysis using RDP4 software (Martin et al. 2010) revealed the clones represented as the HBV/A2 and the HBV/G/A2 recombinant strain (Fig. 1a, b). These findings indicated co-infection of HBV/A2 and HBV/G/A2. 36 nt insertion in the core region and two stop codons in the precore region were not detected in the recombinant strain because two estimated recombination junction of HBV/G were from approximately nt.192 to nt.1795 (Fig. 1b). The HBV/A2 and the HBV/G/A2 recombinant strain were compared with other registered strains including G/A recombinants by MEGA6. Figure 2 shows the phylogenetic tree of these strains. Our HBV/G/A2 were located near the clusters of HBV/G and G/A recombinants. Our HBV/A2 belonged to the HBV/A2 cluster.

The HIV-RNA and CD4 T cell count were 4300 copies/mL and 226 cells/μL, respectively. The HIV subtype was CRF-01 AE and no primary mutations associated with drug resistance mutations were detected. Laboratory findings were as follows: platelet count, 10.6 × 10^4^/dL; asparate aminotransferase, 84 IU/L; alanine aminotransferase, 122 IU/L; alkaline phosphatase, 272 IU/L; γ-glutamyltranspeptidase, 34 IU/L; hepatitis B surface antigen (HBsAg), 93,297 IU/mL; antibodies to the HBsAg (anti-HBsAg), negative; total hepatitis B core antibodies, 11.9 S/CO; HBeAg, 42.9 S/CO; antibodies to the HBeAg (anti-HBeAg), negative; HBV-DNA, >9.1 log copies/mL; hyaluronic acid, 124 ng/mL; Type IV Collagen 7 s, 7.3 ng/mL. HBV drug resistance mutations related to lamivudine, adefovir, entecavir or tenofovir were not detected. He received antiretroviral therapy (ART) consisting of tenofovir/emtricitabine (TDF/FTC) and raltegravir to treat HIV-HBV/G/A2 recombinant infection. Three months after the initiation of ART, while the HBV-DNA decreased steadily, the levels of liver enzyme increased progressively with the rapid decline of HBeAg and HBsAg. The CD4 T-cell count increased to 487 cells/μL. This presentation was consistent with HIV-HBV immune reconstitution inflammatory syndrome (IRIS). The levels of liver enzymes improved without specific treatments. The HBV-DNA decreased to 2.5 log copies/mL after 24 months of ART.

## Discussion

HIV infection accelerates chronic hepatitis B related liver diseases, and precise assessment of HBV infection is imperative in the management of HIV-HBV co-infected patients. Different HBV genotypes induce different clinical features in HBV-infected patients. Recently, acute HBV infection in metropolitan areas of Japan has been increasingly attributed to the HBV/A2, that is transmitted through sexual contacts (Tamada et al. 2012). Another study in Japan found HBV/A2 is the most frequent in HIV infected patients and is detected almost exclusively in homosexual patients (Yanagimoto et al. 2012). Several HBV/G/A2 recombinant strains have been reported among MSM patients in Japan (Tsuzuki et al. 2015; Kojima et al. 2015) and all of these strains have a 36-nt insertion of the core gene. However, the insertion was not detected in the HBV/G/A2 recombinant from our patient, suggesting a possibility that he was infected with the strain outside Japan. The co-infection with HBV/A2 and HBV/G/A2 recombinant was a rare case in HIV-infected patients. The HIV subtype of this patient is CRF_01 AE, which is a prevalent strain in South East Asia (Hemelaar et al. 2006). Hence, we speculated he had been infected with HIV-HBV in Thailand. However, epidemiological data regarding the prevalence of HBV/G or recombinant forms among HIV-infected patients are scarce in Asia (Araujo 2015).

There was a discrepancy between results of the HBV-DNA genotyping and the serological genotyping test by EIA in this case. The reason was unclear, but recombination in the preS2-region might affect the results of the serological test by EIA that used monoclonal antibodies of a combination of epitopes on preS2-region products. HBV/G may be overlooked if a tool designed to detect recombinant forms with other genotypes is not used. It is assumed that detection technique could affect the epidemiologic study of HBV genotype. Full-genome sequence was occasionally difficult to be determined because of the presence of mixture of multiple variant with different length.

Two studies suggest that HBV/G may accelerate liver fibrosis progression and associated with HBeAg positivity and high HBV-DNA levels (Lacombe et al. 2006; Dao et al. 2011). The clinical findings in this case showed the advanced liver fibrosis. Recombination with HIV-HBV/A2 co-infection, which was frequently associated with high HBV-DNA levels, would explain his extremely high HBV-DNA level. Some studies revealed co-infection with HBV/A2 enhanced HBV/G replication (Dao et al. 2011; Kato et al. 2002a, b; Tanaka et al. 2008) and a in vivo study demonstrated the introduction of the HBV/A2 core promoter or core protein or both genomic regions into the HBV/G genome increased HBV/G replication efficiently (Tsuzuki et al. 2015; Sakamoto et al. 2013). Meanwhile, his CD4+ cell count was maintained at 226/μL, nevertheless HIV-HBV-IRIS occurred after the initiation of ART and HBV-specific CD8+ cell responses could be impaired. Thereafter, the impaired cellular immunity caused by HIV infection may affect the progress of liver fibrosis. In contrast, a recent study reports HBV/G does not have a harmful effect on fibrosis progression in efficiently treated HIV–HBV co-infected patients (Calin et al. 2013). Prognosis of chronic hepatitis caused by HBV/G or recombinant forms remain controversial. Long-term precise observation of clinical course is needed to evaluate the prognosis of this patient.

Clinical data regarding treatment for HBV/G is limited. As a rule, HIV-HBV co-infected patients regardless of HBV genotype receive TDF/FTC-containing ART. A study reported three of six patients who experienced HBV-DNA rebound with correct TDF serum concentration were infected with HIV-HBV/G or HIV-HBV/G/A2 mixture (Karine Lacombe et al. 2009). Another French report showed 15 % (7/45) of HIV-HBV co-infected patients who added TDF to lamivudine therapy experienced delayed decrease in plasma HBV-DNA levels, of whom four of them were infected with HBV/G (Lada et al. 2012). A recent report from Japan showed HBV/G/A2 recombinant did not always cause rapid progression of disease (Kojima et al. 2015). In this present case detectable HBV-DNA levels have lasted for 2 years after the initiation of antiviral therapy. Clinical and virological factors to affect TDF/FTC response to HBV/G remained to be determined. Additionally, a case of occult infection with HBV/G/A2 recombinant following acute hepatitis B caused by HBV/A2 is reported (de Barros et al. 2015). Co-infection with HBV/G/A2 recombinant could be a risk of chronic infection caused by HBV/A2 and persistent viremia.

## Conclusions

This patient was infected with HBV/A2 and HBV/G/A2 recombinant which is extremely rare in Japan. Although these HBV strains had no mutations related to anti-HBV drugs, detectable HBV-DNA levels have lasted for 2 years under TDF/FTC-containing ART. Clinicians should keep the HBV genotyping in mind using tools designed to detect recombinant forms in the management of HIV-HBV co-infected patients.

## Abbreviations

HBV: hepatitis B virus
HIV: human immunodeficiency virus
ART: antiretroviral therapy
TDF/FTC: tenofovir/emtricitabine
IRIS: immune reconstitution inflammatory syndrome
